# Overview of lasers

**Published:** 2008-10

**Authors:** Uddhav A. Patil, Lakshyajit D. Dhami

**Affiliations:** LakshyaKiran Cosmetic and Laser Centre, Kolhapur, Maharashtra, India; 1 Vasudhan Arjin Cosmetic and Laser Surgery, Mumbai, Maharashtra, India

Laser surgery of skin conditions having cosmetic implications has revealed the profound psychological benefits which are unmatched by any other modality of treatment either with or without a knife. An increasingly sophisticated understanding of the biophysics of laser-tissue interactions has lead to a more efficient utilization of the present technology on the clinical side and at the same time is helping the physicists to add more and more highly selective laser systems in the armamentarium of aesthetic laser surgeons.

This article provides a general overview of lasers in skin and cosmetology and discusses its current clinical applications from Plastic Surgeon's point of view.

## HISTORY

*Laser* is an acronym for “Light Amplification by Stimulated Emission of Radiation.” Stimulated emission was based on Einstein's quantum theory of radiation..[1] The first laser was produced by Theodore H. Maiman on 7^th^ July 1960 using ruby as a lasing medium that was stimulated using high energy flashes of intense light.[2] The Decade of 1960s will always be remembered in the history of lasers as more than ten different lasers were invented using solid, gaseous, semi-conductor as well as liquid lasing media. The refinement of technology along with invention of newer lasers has continued till date and will keep on doing so in the future as well.

A significant understanding of lasers and light sources is required for their optimal use. Also a basic understanding of laser physics is mandatory to carry out an efficient laser treatment.

## LASER CHARACTERISTICS

Laser light is monochromatic, bright, unidirectional and coherent.

### 

#### Monochromaticity:

The luminous waves emitted come out with the same wavelength and energy. A single wavelength or a narrow band of wavelengths emitted allows precise targeting within tissue, while sparing adjacent structures.

#### Brilliancy:

The light beam emitted is extremely intense and angularly well centred. The brightness or intensity is one of the important properties and can be enhanced by techniques like pulsing and Q-switching where extremely high peak power can be delivered in nanoseconds.

#### Coherency:

All the photons emitted vibrate in phase agreement both in space and time. Coherence is a measure of precision of the waveform. Highly coherent laser beam can be more precisely focused.

#### Directionality:

All the photons travel in Uni direction. Directionality of the laser correlates with the emission of an extremely narrow beam of light that spreads slowly. Within the laser apparatus, efficient collimation of photons into a narrow path results in a divergence factor of approximately 1 mm for every metre travelled. Directionality allows the laser beam to be focused on a very small spot size.

### Terminology and measurements

1. IPL: Intense Pulsed Light where peak optical power per pulse is up to 20,000 watts achieved with capacitor banks. All bright light sources are not called IPL, they are just light sources. Wavelengths emitted range usually from 400 nm to 1200 nm and the lower wavelengths can be eliminated by various cut off filters which usually range from 515 to 755 nm.
2. I^2^PL: Second generation Intense Pulsed Light where wavelengths from 900 to 1200 nm are eliminated.
3. Chromophore: Chromophore is a material, present either endogenous in the tissues or exogenous i.e. brought from outside, which absorbs particular wavelengths depending on its absorption coefficient. Examples of endogenous chromophores are melanin, haemoglobin, (oxy haemoglobin, de-oxyhaemoglobin and meth haemoglobin), water, protein, peptide bonds, aromatic amino acids, nucleic acid, urocanic acid and bilirubin.[3] Exogenous compounds like different colors of tattoo ink also act as chromophores.
4. Parameters: Parameters are the values of wavelength, fluence (see below), number of pulses, pulse duration, pulse delay, repetition rate and spot size which are set on laser or IPL systems to treat a particular condition.
5. Lasing is the process of treating a lesion or a condition with lasers or light.
6. Wavelength: The distance between two subsequent peaks or troughs of a light wave. Usually it is expressed in nm (nanometre i.e. 10^−9^ metres)
7. Hertz (Hz): A unit of frequency equal to one cycle per second.
8. Frequency (V or f) ∝ (1/ wavelength (Hz) Therefore shorter the wavelength, higher is the frequency and longer the wavelength, lower is the frequency.
9. Photon: Photon is an elementary particle responsible for electromagnetic phenomena. It is the carrier of electromagnetic radiation of all wavelengths, including in decreasing order of energy, gamma rays, X-rays, ultraviolet light, visible light, infrared light, microwaves, and radio waves. The photon differs from many other elementary particles, such as the electron and the quark, in that it has zero rest mass; therefore, it travels (in a vacuum) at the speed of light.
10. Energy: Each photon carries a ‘quantum’ of energy (E), whereby: E=hV (h – Planck's constant) Therefore:
  Short wavelength = high frequency = high energy photons
  Long wavelength = low frequency = low energy photons

Measurements used routinely in laser applications include wavelength, frequency, energy, fluence, power, and irradiance.

1. Energy: Energy is measured in joules (J) and is proportional to the number of photons.
2. Power: Power is the rate of delivery of the energy. It is measured in watts (W) where 1 W = 1 J/sec.
3. Fluence: Fluence is the energy delivered per unit area. It is measured in J/cm^2^
4. Irradiance: Irradiance is the power per unit area. It is measured in W/cm^2^

## LASER TISSUE INTERACTION[3]

Laser beam that encounters skin surface may be reflected, transmitted, scattered or absorbed at each layer. Once the laser beam falls on the skin, from this point onwards, we should think about it not as a light but as a continuous or pulsed source of photons. Photon as a particle can only interact with matter by transferring the amount of energy. Therefore, only absorbed photons can produce tissue effect. For photon absorption in a tissue, a chromophore is required. Therefore, our aim is to increase the photon absorption by reducing its reflectance, scattering and transmission.

### 

#### Transmission:

If there is no chromophore then all the photons will pass through the tissue without producing any effect. This is total transmission. Therefore, selection of a proper chromophore in or near the target tissue is a first important step in laser therapy.

#### Reflection:

Reflection occurs at all interfaces of media through which the laser beam is travelling, such as optical glass or sapphire tip, air, water jelly and skin surface. For example, the stratum corneum reflects approximately 4% to 7% of visible light that encounters the skin surface. Reflection is minimized by either a firm contact between the laser head of contact lasers or a light guide of I^2^PL system and skin or by using a layer of optically transmissible clear jelly between them in case of IPL systems.. In case of focused and collimated beams, reflection can be minimized by holding the hand piece exactly perpendicular to the skin surface.

#### Scattering:

Scattering is due to lack of homogenety in the skin's structures, such as molecules, organelles, cells or larger tissue structures. In the dermis, scattering has been shown to occur predominantly from inhomogeneities in structures whose size is of the order of the wavelength or slightly larger e.g. collagen fibres. It, therefore, appears to act as a turbid matrix in which scattering is an approximately inverse function of wavelength (shorter wavelength, greater scattering). The greater the scattering, less will be the depth of penetration, and more possibility of absorption.[4]

In the tissues more scattering occurs when a small spot is used. In case of large spot, after scattering, photons hit each other and get recollected and redirect themselves in the direction of the beam thereby increasing the depth of penetration. Therefore, larger the spot deeper is the penetration. However the selection of spot size also depends on the power generation of the system as well as on the depth of the chromophore from the skin surface.

Absorbed photons can produce thermal, mechanical, or chemical changes in and around the chromophore. Out of these, thermal changes are most useful, as in hair reduction, skin rejuvenation and in vascular lesions. Physical or mechanical tissue changes, known as photoacoustic changes occur when high energy photons are delivered in ultra short pulses of nanoseconds. This is made use of in removing tattoos and clearing certain pigmented lesions.

Example of light energy causing a chemical reaction is photosynthesis in plants. The UV irradiation induced chemical changes in deoxyribonucleic acid (DNA) may be responsible for cell death and neoplastic transformation.

### Selective photothermolysis

In 1983, Anderson and Parrish[5] described the theory of selective photothermolysis, which revolutionized laser therapy by explaining a method of producing localized tissue damage sparing the surrounding tissues. For understanding this we need to understand certain terms.

1. Threshold fluence of a tissue is a fluence, which if equaled or exceeded leads to the tissue destruction.
2. Thermal relaxation time (TRT) is defined as the time required by an object to cool down to 50% of the initial temperature achieved.

For tissue damage to ensue a wavelength should be preferentially absorbed by the chromophore in the target tissue and not absorbed by the surrounding tissue, it therefore needs to be delivered in a pulse duration which is less than or equal to the thermal relaxation time (TRT) of the target. If delivery time exceeds the TRT then the target does not get damaged instead the energy dissipates to the surrounding tissues inflicting injury there. Even if energy delivery occurs within the TRT limits, the fluence reaching the target after subtracting reflection and scattering in the path needs to equal or exceed the threshold fluence to cause tissue destruction. This seemingly difficult task can be achieved by manipulating three variables: wavelength, pulse duration, and fluence.

#### Wavelength:

**Figure 1:**
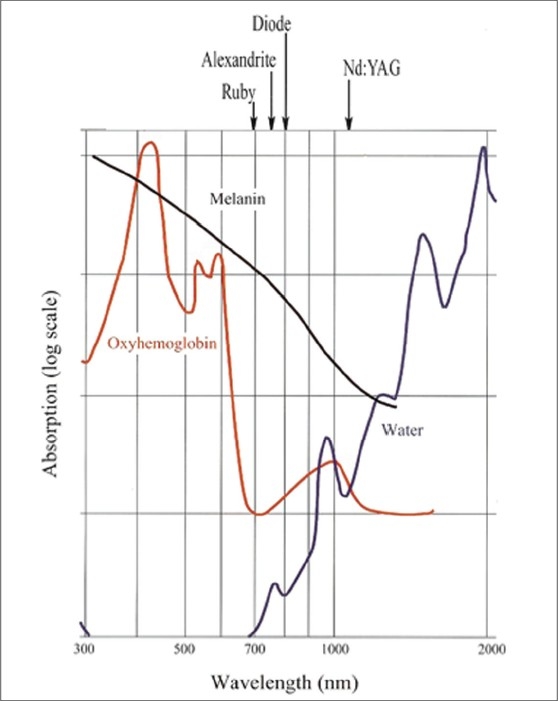
The absorption of various chromophores as a function of wavelength.[6]

Wavelength has a dual impact attributable to its absorption coefficient in different chromophores [Figure 1][6] as well as the depth of penetration from the skin surface, which roughly increases as the wavelength increases in the visible and near infra-red spectrum. After carefully choosing a proper wavelength for a particular chromophore, the laser surgeon has a difficult task of delivering maximum number of photons to the target chromophore before they are snatched by competing chromophores which are present before the target. By manipulating the other two variables i.e. pulse duration and fluence, this can be done effectively. Not only this, but by choosing proper pulse duration a smaller or a larger target having the same chromophore can be selected.

#### Pulse duration:

Selection of pulse duration is mainly guided by the TRT, which in itself is related to the size of the target. As a general rule, larger the chromophore, longer is the TRT as large objects take long time to cool. More difficulty is encountered while lasing the smaller targets as the short pulse width required makes the epidermis full of melanin more vulnerable (TRT 10 ms). This is achieved by dividing this short pulse into still shorter rations of pulses which are delivered in succession. This form of energy delivery is called multiple synchronized pulsing.

#### Multiple Synchronized Pulsing:

**Figure 2:**
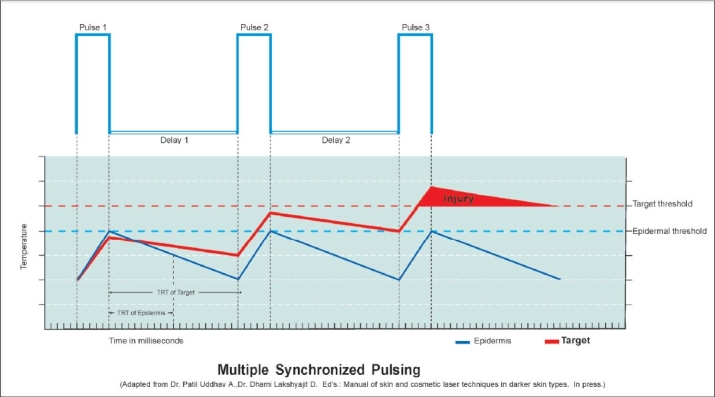
Multiple synchronized sequential pulsing.[7]

[Figure 2] Even though the epidermis is a strong competing chromophore for smaller targets, it can be spared as long as the TRT of the target is longer than that of epidermis. Longer TRT means the target takes longer time to cool to 50% of the temperature achieved. By multiple synchronized pulsing, which are usually two to three and maximum five pulses, with delays in between, both the target and epidermis are heated by the first pulse just within the threshold limit of the epidermis, therefore the epidermis is not damaged. After the first pulse there is a delay, during which both epidermis and target start cooling, but as the target cools slowly at the end of the delay it still retains some heat while the epidermis has cooled down completely. During the second pulse and the delay thereafter, the same sequence repeats but now the target gets heated to a higher level as it is starting from an elevated base line. This pulsing is repeated till such time as the temperature in the target at the end of last pulse exceeds its threshold limit.[7]

## CLINICAL APPLICATIONS

The present clinical applications in skin conditions and cosmetology which most concern a plastic surgeon can be grossly divided into following five categories:

1. Unwanted hair
2. Vascular lesions, acne and scars
3. Pigmented lesions and tattoos
4. Skin rejuvenation by ablative and non ablative laser resurfacing
5. Leg veins and varicose veins

### Unwanted hair

During the last decade permanent reduction of unwanted hair by lasers and light sources has been established as a quick, safe and sure method of choice over all earlier modalities of hair removal which were temporary. For permanent reduction the hair root should be destroyed. The term hair root refers to hair within the hair follicle. Here it will be appropriate to quickly review the relevant anatomy.

### Hair follicle anatomy

Hair follicle is divided into three units:

*Infundibulum* includes the region from the hair follicle orifice to the sebaceous duct entrance.

*Isthmus* encompasses the region between the entrance of the sebaceous duct and the attachment of the arrector pili muscle.

*Inferior segment* is the region from the insertion of the arrector pili muscle to the base of the follicle and includes the hair follicle bulge and the bulb.

*The hair follicle bulge* is the extended lower portion of the hair follicle between the insertion of the arrector pili muscle to the hair bulb. The bulge has more or less constant distance of 1.5 mm from the skin surface.

*The hair follicle bulb* is the lowest portion of the hair follicle and is composed of matrix cells, interspersed by melanocytes, which produce hair. The distance of the bulb from the skin surface varies with the different stages of hair growth. It is shallowest at early anagen and deepest at late anagen stage. In anagen itself coarser the hair, deeper will be the follicle [Table 1].

**Table 1:** Corresponding hair shaft diameter with anagen hair follicle depth. (Exceptions exist)

Quality of the hair	Shaft diameter microns	Anagen follicle depth Millimeters from skin surface
Very fine	< 25	< 1
Fine	25-51	1-2
Medium	51-76	2-3
Coarse	76-102	3-4
Very coarse	102-127	4-5
Extra coarse	127-152	5
Super coarse	> 152	5

Melanin of hair root is the chromophore which absorbs photons and gets heated. Melanin has a wide and gradually sloping down absorption coefficient curve spanning from ultraviolet to infrared spectra giving a wide choice of wavelengths to chose from [Figure 1]. This liberty of choosing wavelengths is restricted by the presence of the same melanin as a competing chromophore in variable quantities in the epidermis of different skin types. An ideal target is an extra coarse, dark black hair root in anagen growth phase on a lighter skin type. The most difficult is a fine, lightly pigmented hair of a darker skin type. In any case, if the target is lighter than the epidermis, no available laser can act on it at present. However, attempts are underway to enhance the absorbability of such lighter targets by incorporating external pigment in them. Meladine™[8] is one such exogenous chromophore enhancer.

Hair root is one example where target and chromophore are not exactly the same. Melanin content of the hair bulb and bulge is the chromophore which absorbs photons and gets heated. The target is follicular epithelium which is at some distance surrounding the bulb. Therefore, the bulb needs to be heated long enough to allow sufficient heat to get conducted to the target. Though the chromophore in case of extra coarse, medium and fine hairs is melanin, they can not be lased with the same parameters as the size of the chromophore in the bulb and bulge is not the same in three. The root of an extra coarse hair will never get destroyed, however high the energy, if you use a shorter pulse. You are just not allowing sufficient time for a larger chromophore to get sufficiently heated and dissipate its heat to the surrounding target. Extra coarse hair needs longer pulse duration than medium, and fine hair needs the shortest. Therefore, the pulse duration should be primarily guided by the size of the chromophore and only secondarily by the competing chromophore. Epidermal melanin poses as a competing chromophore in case of all lasers in the visible and infra red spectrum of light [Figure 1], therefore it will be worth to consider a classification of skin based on epidermal melanin concentration and its response to sun [Table 2].

**Table 2:** Fitzpatrick's skin photo types[9]

*Skin type*	*Skin colour*	*On exposure to sun*		*Lancer ethnicity scale*
		
		*Burns*	*Tans*
Type I	White	Always	Never	Caucasian
Type II	White	Usually	With difficulty	Caucasian
Type III	White	Sometimes	Averagely	Caucasian
Type IV	Moderate brown	Rarely	Easily	Asian, mid eastern
Type V	Dark brown	Very rarely	Easily	Indian
Type VI	Black	Never	Very easily	Black

Shorter wavelengths like Ruby 694 nm and Alexandrite 755 nm were the first ones to be used for hair reduction. Though they have higher affinity to melanin, they can not penetrate deeply in the skin. In darker skin types V and VI they led to unacceptable rate of complications like epidermal burns and post treatment hyper pigmentation due to strong competing epidermal melanin. In contrast longer wavelengths like Diode 800 nm and Nd:YAG 1064 nm though having lesser affinity to melanin as compared to shorter ones are found to be more effective due to deeper penetration to the deeply placed hair follicles which at times are 3 to 4 mm deeper. At present both these wavelengths are found effective for hair reduction in darker skin types. Therefore the reach of the wavelength to the target is also an important consideration. Remember, photons have to first reach the target to get absorbed.

### Vascular lesions, acne and scars

In vascular conditions, haemoglobin is the chromophore and the vessel wall is the target. Water contained in the cells of the vessel wall also acts as an additional chromophore. Haemoglobin has absorption coefficient curve with peaks at 418 nm, 524 nm, 577 nm and again at 1064 nm [Figure 1]. At the first and second peaks the melanin absorption is also very high and therefore these wavelengths can not be used. However, the spectrum between 580 nm and 590 nm is useful clinically. Pulsed dye laser (PDL) with a wavelength of 595 nm is at present considered as a gold standard for vascular lesions. Some advanced systems have Dynamic Cooling Device (DCD™).[10] cooling to safeguard the epidermis. Here a cryogen spray falls on the skin milliseconds before the laser pulse and cools the epidermis by rapid evaporation thus protecting it.

#### Port wine stains (PWS)

**Figure 3:**
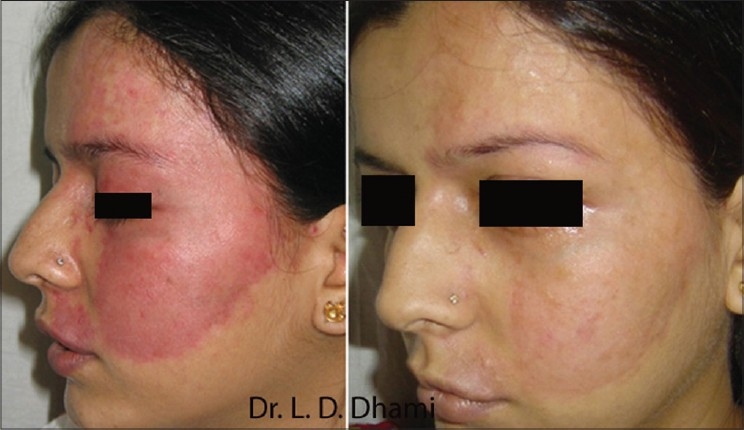
PWS before and after 6 treatments with PDL 595 nm

**Figure 4:**
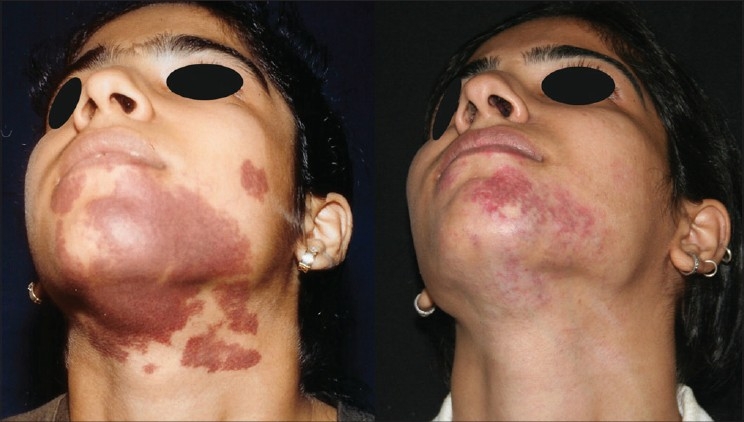
PWS before and after 10 treatments of IPL with 590 nm filter

Flash lamp pumped dye laser was the first example of a laser which was specifically built to treat PWS after understanding the principles of selective photothermolysis having 577 nm wavelength to match 3^rd^ peak of the absorption curve of haemoglobin and 450 micro second pulse duration which was less than the TRT of the smaller vessels of PWS. Later on it was replaced by yellow light at 585 nm and 595 nm having deeper penetration and giving better results. PDL treatment is the method of choice for most PWS in children [Figure 3]. The IPL sources with a cut off filter of 590 nm are also quite effective and are being routinely used for a variety of vascular lesions including PWS [Figure 4].

By extending the principles of selective photothermolysis and by making use of multiple synchronized pulsing, the laser surgeon is now able to selectively target a larger or a smaller vessel in the same lesion having the same common chromophore haemoglobin. The only limiting factor in laser treatment of vascular lesions at present is the reach of the lesion (i.e. its depth from the skin surface. Currently a wavelength of 1064 nm can reach a maximum of 8 mm) and the flow in the lesion (only slow flow lesions can retain the generated heat long enough for the tissue destruction).

#### Haemangioma

**Figure 5:**
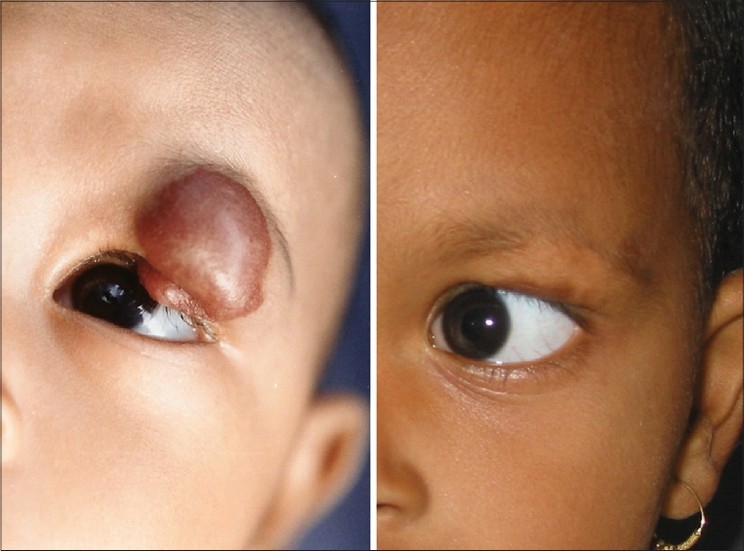
Haemangioma in a five month old baby in rapid proliferative phase before and 1 year after 5 treatments with Nd:YAG 1064 nm. Note undamaged eyebrow and eyelashes, example of chromophore specificity. Eye was protected by a special metal corneal shield.[11]

The commonly used 590 and 595 nm wavelengths, being shorter, can act only on superficial lesions up to 1.2 mm depth. PWS, superficial haemangiomas and other superficial vascular lesions are treated by these. For deeper vascular lesions like deep haemangiomas, 1064 nm wavelength is used [Figure 5].

#### Acne

**Figure 6:**
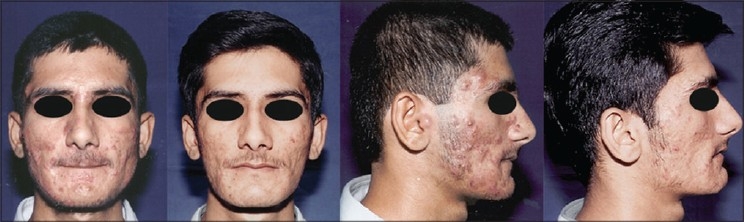
Severe pustular acne before and after 2 IPL treatments (590 filter) as a part of multimodality approach

Non intense Light Heat Energy (LHE) sources, IPLs and Lasers are playing an important role in multi-modality approach to management of acne and at present their role is three fold: in active acne with haemoglobin and water as chromophores, [Figure 6] in acne induced pigmentation and in post acne scarring. Diode 1450 nm with water absorption is found effective in inflammatory acne. This as well as Erbium: glass 1540 nm Lasers are found promising in contouring atrophic acne scars.

#### Scars

**Figure 7:**
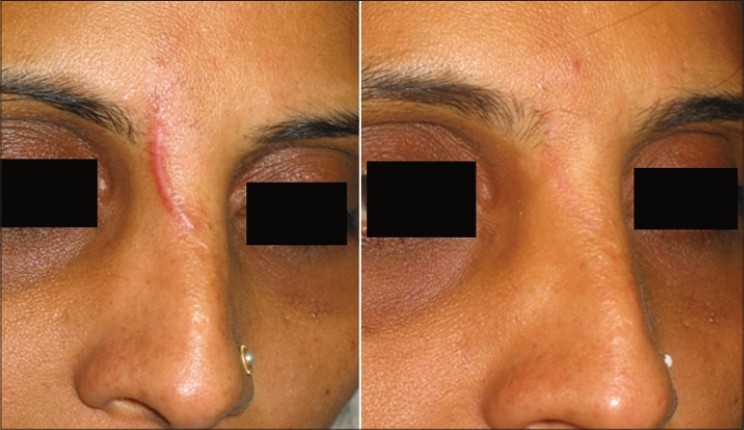
Post traumatic hypertrophic scar before and after 1 PDL and 4 IPL treatments (590 filter.)

**Figure 8:**
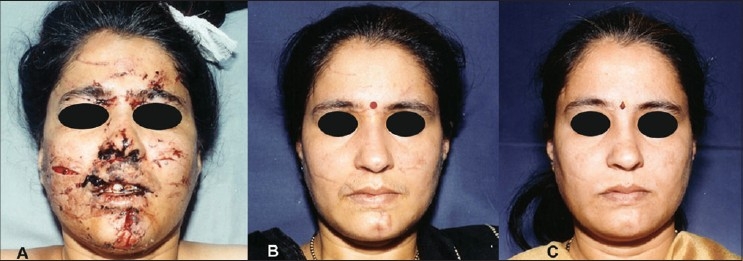
(A) Extensive windshield injury of face. (B) Result after primary plastic surgical repair. (C) Result after 4 IPL treatments (590 filter) at monthly interval

In scars role of lasers and IPL systems spans from fibroblast modulation in fresh scars, clearing scar pigment, removing traumatic tattoos to remodelling of collagen in hypertrophic scars [Figure 7] and even older scars. Fresh scars are hypervascular, haemoglobin is the abundant chromophore and fibroblast is the target. Fresh scars can be made inconspicuous. PDLs, IPLs with 590 cut off filter have shown good results [Figure 8]. Recently introduced Er: Glass 1540 nm fractional lasers also have shown promising results.

### Pigmented lesions and tattoos

As melanin absorbs light at a wide range of wavelengths, from 250 nm to 1200 nm [Figure 1] almost any Laser with sufficient power causing thermal denaturation can be used to remove benign pigmented lesions of the epidermis. In dermal pigmented lesions the target chromophore is intracellular pigment melanosomes or tattoo particles. To make such sub-micrometer particles absorb photons, the energy delivery needs to be in nanoseconds (TRT) as compared to milliseconds for hair root and microseconds for capillaries. This has been made possible by a technique invented in 1962 called quality switching or Q-switching. Normally in a lasing chamber, once population inversion occurs i.e. the number of stimulated photons exceeds resting photons, the laser beam emerges through the partially reflective mirror. In Q-switching, the energy or number of stimulated photons is deliberately allowed to build up to a higher level by blocking the partially reflective mirror with a Pockels cell. Pockels cell contains laminar opaque crystals which become transparent only when an electric current is applied for a few nanoseconds controlled by an electronic switch. When transparent, the built up photons get released just for a few nanoseconds and blocked again to get rebuilt up. These ultra short high energy bursts of pulses lead to mechanical photo acoustic damage in the target cells. Q-switching is available with Ruby 694 nm, Alexandrite 755 nm, Nd:YAG 1064 nm and frequency doubled FD Nd:YAG 532 nm (i.e. in the path of QS Nd:YAG 1064nm beam, a KTP crystal is brought which changes the frequency to double so the 1064 nm wavelength becomes half i.e. 532.) KTP laser is also available as a non QS laser with 532 nm wavelength. This is an example of a laser beam being used as a source of energy (instead of a flash lamp) to stimulate another lasing medium. The rate of heating and rapid material expansion with Q-switched Lasers can be so severe that tissues are torn apart by shock waves, cavitation or rapid thermal expansion. The immediate effect on the pigmented skin is whitening, due to melanosome rupture and pyrolysis leading to formation of gas bubbles that scatter light. Immediate whitening offers a clinically useful treatment end point.

Selective photothermolysis with various Lasers is highly useful for epidermal and dermal pigmented lesions in which cellular pigmentation is the cause, however lasers and IPLs have variable usefulness for dermal melasma and post inflammatory hyper pigmentation because the etiological factors may still persist and lead to recurrence.

#### Treatment

**Figure 9:**
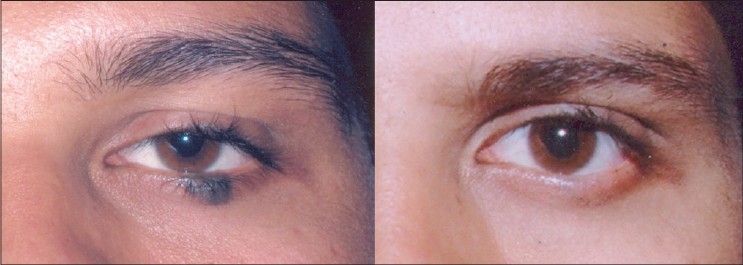
Congenital melanocytic nevus before and after 8 IPL treatments. First 6 treatments with 645 filter for deeper pigment and last 2 with 615 filter for superficial pigment. Eye was protected by a special metal corneal shield.[11]

**Figure 10:**
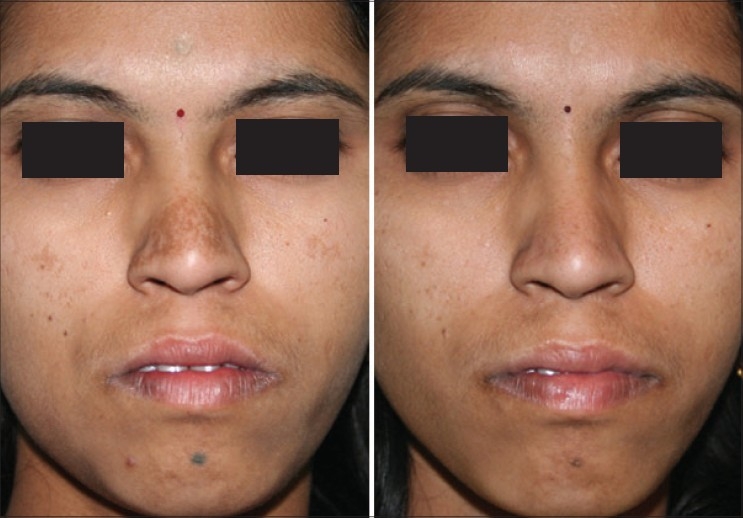
Melasma before and after 2 IPL treatments with 645 filter. QS Nd:YAG 1064 nm has cleared residual forehead tattoo around a punch graft in one treatment and has significantly lightened the dark tattoo over chin, freckles and moles with two treatments

The approach to the treatment depends on location of pigment (epidermal, dermal or mixed), the way it is packaged (intracellular or extracellular) and the nature of pigment (melanin or tattoo particle). Continuous wave lasers like CO_2_ (10,600 nm) or Er-YAG (2940 nm) with water as a target chromophore in epidermis can be used for removing the superficial pigmented skin, especially seborrheic keratosis and ‘Q-switched resistant’ Café-Au-Lait macules. But the non selective thermal injury may lead to some erythema and possible pigmentary and textural changes. In many pigmented lesions however, the melanosomes and melanocytes are clustered so compactly that they act as a larger body of chromophore. In this situation, melanin specific wavelengths even in millisecond domain also lead to lesion clearance. These long pulsed lasers are only suitable to treat nevocellular nevi while IPL (500 nm to 1200 nm) treats photo damaged pigmentations like solar lentigines, dyschromia and melasma [Figures 9,10].

**Figure 11:**
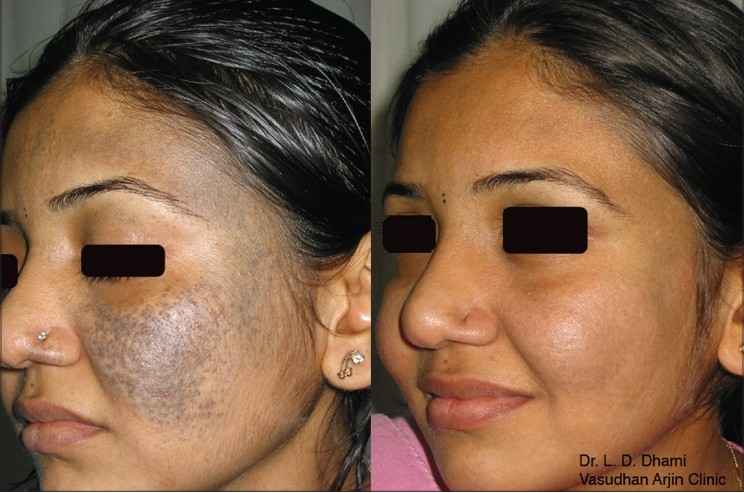
Nevus of Ota before and after Q-Switched Nd:YAG 1064 nm laser treatments. Please note the complete clearance of the pigment without any residual textural skin change

**Figure 12:**
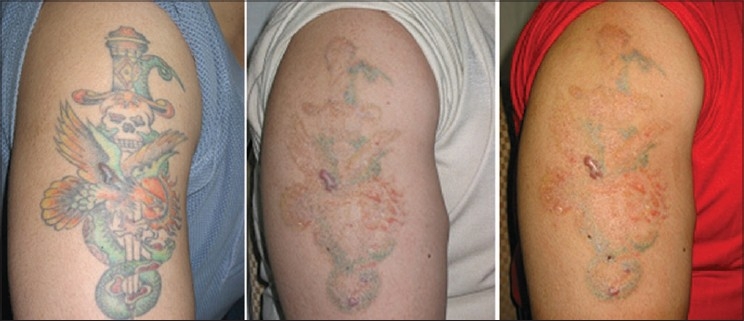
Multicolored tattoo treated with 4 simultaneous treatments with Q-Switched 1064 nm (for black and green) and 532 nm (for red). Small hypertrophic scar is pre-existing

**Figure 13:**
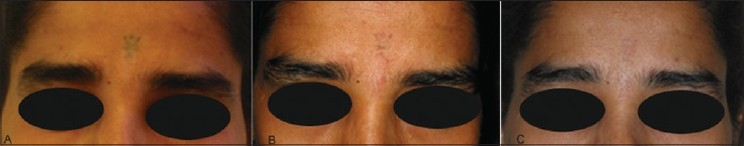
Forehead tattoo cleared with 3 sittings of Q-Switched Nd:YAG 1064 nm. (A) After 1st Treatment (B) After 2nd Treatment (C) After 3rd Treatment

The Q-Switched Lasers (532 nm FD 694 nm Ruby, 755 nm Alexandrite or 1064 nm Nd-YAG) with their high peak power and pulse width in nano second range are best suited to treat various epidermal, junctional, mixed and dermal lesions The Q-switched Nd-YAG is an ideal choice to treat dermal pigment as in nevus of Ota and in darker skin types, as it reduces the risk of epidermal injury and pigmentary alterations. The pigment clearance may be expected to be near total [Figure 11]. In case of tattoos however, no other laser technique is found to be more effective than Q-switching. In a multicolored tattoo for black ink QS 1064 nm, QS 755 nm and QS 694 nm are excellent. For red ink FDQS 532 nm is excellent. For green ink QS 755 nm, QS 694 nm and QS 1064 nm are found to work in that order. [Figure 12]. In India most commonly done tattoos are amateur and religious using carbon which clears very well with 1064 nm. [Figure 13]

#### Treatment pearls

Local anaesthetic cream is applied 1 hour before treatment to reduce pain and discomfort. The desired tissue response with Q-switched Lasers is immediate whitening of treated area and none or minimal pin point bleeding. It is best to use largest spot size with optimum power to prevent epidermal damage. Post treatment care consists of antibiotic cream with or without steroid for a few days, avoidance of sun exposure with use of sun block creams and bleaching creams at night to prevent laser induced hyper pigmentation. Multiple treatment sessions are preferred at 6 weeks or longer interval with higher fluence for subsequent treatments.

### Skin rejuvenation by ablative and non ablative laser resurfacing

Resurfacing Laser techniques were developed for their use in Tissue Tightening for wrinkles and rhytides, Skin Lightening for superficial sun damage dyschromia, lentigines or actinic keratosis and Skin Leveling for atrophic acne or chicken pox scars.

#### Ablative resurfacing

Ablative resurfacing with CO_2_ laser (10,600 nm) was the only option during mid 1990s, with either continuous wave or ultra short pulse mode, with or without a scanner. These lasers proved very effective in the Plastic Surgeon's armamentarium, but were found to have significant side effects, especially in inexperienced hands. Prolonged erythema, hyper pigmentation, contact sensitivity to topical products, infection, prolonged hypo pigmentation and deep scarring were main concern.

To decrease these side effects Er:YAG (2940 nm) laser with stronger water absorption and reduced deeper thermal damage came as an advancement. But though Er:YAG laser was very good for skin lightening and levelling, the tissue tightening effect was much less than with CO_2_. Increasing the pulse width increased the residual thermal damage providing additional benefit but also increased side effects. The next development came as a laser that combined Erbium with CO_2_. Erbium provided pure ablation, while CO_2_ allowed heat deposition for skin tightening. Use of topical anaesthetics, which hydrated the skin also helped minimise side effects. Despite all these advances, ablative lasers lead to significant down time of few weeks to months. The common side effects seen often are erythema, oedema, infection, flare up of acne, hyper pigmentation, relative hypo pigmentation and scarring. The true contraindications are history of vitiligo, scleroderma, darker skin patients, and unrealistic expectations.

**Figure 14:**
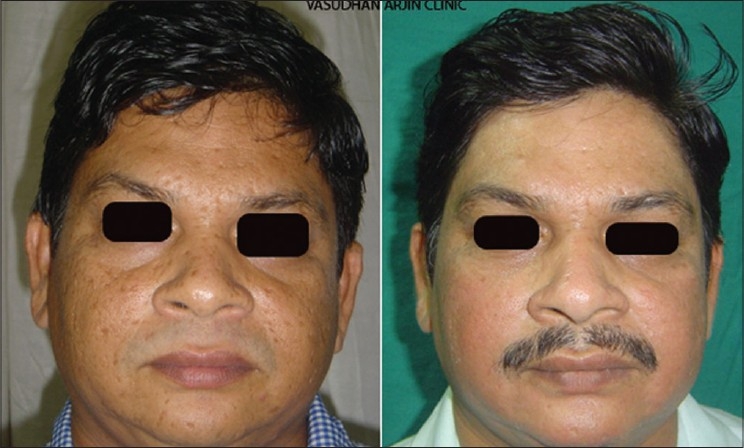
(A) Small pox scars (B) After combined derm abrasion and Er:YAG 2940 nm laser resurfacing in a single sitting

Ablative laser resurfacing leads to dramatic improvement in the overall quality of skin. The gains can be up to 50% or more, including reduction of wrinkles, levelling of deep scars and vaporisation of superficial skin lesions [Figure 14]. Unacceptable down time of this procedure led to the development of a promising non-ablative resurfacing technique, which is now in the forefront.

### Non ablative laser resurfacing

Currently the devices which are available in this field are either with a vascular target or the ones that target water in the dermis. The first group includes pulsed dye lasers (PDL) and intense pulsed light (IPL) devices, which initiates a cascade of events by controlled wounding of the dermal micro-vasculature. The second group includes mid infra red lasers that target water in the dermis to effect dermal heat deposition. This group includes 1320 nm Nd:YAG, 1450 nm Diode and 1540 nm Erbium: glass lasers.

The main advantage of non ablative laser rejuvenation is lack of any downtime for the patients. The devices that target dermal vasculature will help minimise telangiectasias, diffuse erythema or rosacea. Devices which also target melanin like an IPL can also treat lentigines, melasma, poikiloderma, dyschromia and other non specific hyper-pigmentation.

Non ablative resurfacing is best for patients with fine lines, though multiple treatment sessions are usually necessary. The degree of wrinkle reduction may not be as dramatic as seen with ablative lasers, so patient dissatisfaction can be an issue.

**Figure 15:**
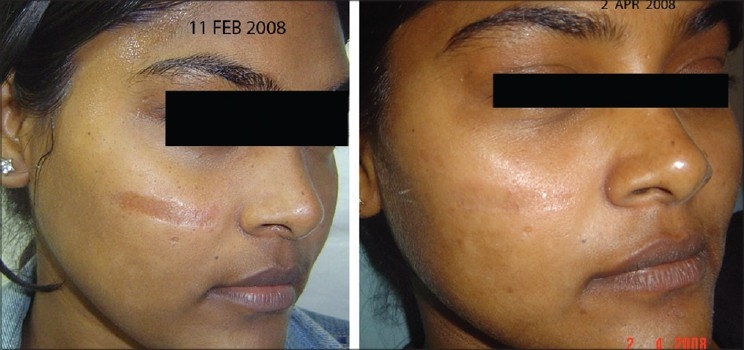
Spread and Atrophic ARSTL scar dramatically improved with Matisse TM[12] Fractional 1540 nm Erbium: glass laser non-ablative resurfacing

The wide array of non ablative rejuvenation lasers allow us to individualise treatment based on the patient's specific concerns. If the vascular element is the main concern then PDL or an IPL with 590 nm filter would be most suitable. If pigment disorder is the main issue then IPL with 645 nm filter or near infra red laser of 1064 nm Nd:YAG would provide the best improvement. The mid infra red lasers induce changes such as vascular damage, apoptosis, water vaporisation in the dermis and oedema with imperceptible wounding, which then leads to a cascade of inflammatory processes and subsequent neo-collagenesis. The laser beam in effect, penetrates up to a depth of 2 mm (papillary dermis), resulting into reduction of rhytides. The different wavelengths of 1320, 1450 and 1540 nm are all often used interchangeably with similar efficacy, though 1450 nm Diode and 1540 Erbium: glass Lasers are superior in the recontouring of atrophic scars [Figure 15] and treatment of inflammatory sebaceous glands. The field of non ablative resurfacing has expanded dramatically over last 10 years, while the ablative laser procedures have remained stagnant during this period.

The efficacy of this laser has been demonstrated by histological evidence, photography and clinical satisfaction with tighter, firmer and more toned skin. This occurs as a result of increase in the number of fibroblasts, increased collagen deposition and normalisation of papillary dermis. The side effects commonly seen are transient pain, oedema and erythema. Pigmentary alteration, blister formation and scarring are very rare.

### Fractional resurfacing

Recently a new technology has evolved, which allows ablative resurfacing of the skin but only on a small fraction of the skin at a microscopic level. The area treated with each pass is not more than 10% of the total surface area. The wavelength used is 1540 nm Erbium: glass with either a scanning device (Fraxel) or an array of micro lenses (Matisse™[12]).

Fractional Laser Resurfacing delivers light in an array of high precision micro beams. These micro beams create narrow, deep columns of tissue coagulation that penetrate well below the epidermis and into the dermis, while sparing the tissue surrounding the columns from damage. The coagulated tissue within the columns initiates a natural healing process that accelerates the formation of new, healthy tissue. Coagulated cellular debris is expelled as the skin is resurfaced.

With the fractional Matisse laser there are 1000 micro spots, in one sq. cm area with each spot diameter of approximately 100 to 125 microns. The depth of penetration of Laser energy is approximately 600 microns to 1 mm. (up to papillary dermis) or more.

Unlike with selective photothermolysis, where the whole of the selected target area is damaged “Fractional Photothermolysis” seeks to only damage certain zones within the selected target area, (producing tiny dot, or pixel-like treated areas on the skin), leaving the other zones within it perfectly intact; hence only causing fractional damage through the heat of the light source. This allows the skin to heal much faster, than if the whole area was treated, as the ‘healthy’ untreated tissue surrounding the treated zones helps to fill in the damaged area with new cells very quickly.

Fractional Photothermolysis or Fractional Laser Skin Resurfacing can therefore be compared to the precise alteration of digital photographs that we are able to do nowadays; pixel by pixel.

The concept of this fractional laser technology can be applied with either ablative laser resurfacing or non-ablative laser skin rejuvenation, using the various different wavelengths of lasers available.

This fractional approach (ablative and non-ablative) claims to achieve comparable skin improvements as obtained with conventional ablative laser resurfacing with an Er:YAG or CO_2_ laser, (depending on depth and severity of wrinkles), but without the associated side effects or downtime; i.e. you get the results of an ablative laser but with the downtime of a non-ablative laser.

Optimal improvement after fractional treatment is usually visible in about 2 - 3 months as the collagen remodelling and skin tightening continues. The longevity of results are comparable to ablative laser resurfacing and as always, is dependent on the future ageing, effects of gravity and sun exposure.

Fractional photothermolysis is used for the treatment known as skin rejuvenation or skin resurfacing. This includes the reduction and possible removal of fine lines and wrinkles, improvement of deeper wrinkles, repair of sun damaged skin on the face, neck, shoulders and hands, the reduction of age spots and blemishes, acne scars and hyper-pigmentation, melasma (areas of darker pigment or brown patches in the skin), telangiectasias and acne rosacea.

Most patients will be able to return to work immediately following this type of procedure. Due to the way the laser treats the skin, it remains relatively strong, therefore any redness can be camouflaged with make-up straight away without any ill effects; and men are also able to shave almost immediately after the treatment.

### Repeat procedures

Unlike with ablative laser resurfacing, where one treatment is usually enough; multiple treatment sessions are required with fractional resurfacing to obtain optimal results. The clinical studies carried out suggest that generally 4-6 treatments spaced about 7 to 21 days apart produce a gradual remodelling of the skin, until complete healing occurs; when old tissue is replaced with fresh collagen and elastin filled tissue. One can expect an overall improvement in the range of 40 to 50%. The number of treatment sessions required depends upon the individual patient and the condition undergoing treatment.

### Adverse effects / Complications

Erythema and oedema is common and resolves within few hours or a day or two. Pigmentary changes either hypo or hyper pigmentation may be visible after few days but usually resolves in 3 to 6 months.

Superficial burn or blister may rarely occur, and can be minimised by careful technique and adherence to the proper protocols

### Leg veins and varicose veins

De-oxyhaemoglobin has almost similar absorption curve as oxy-haemoglobin. The thread veins and some of the smaller veins which are dilated up to 5 mm can be treated with deeply penetrating Nd:YAG 1064 nm

For varicose veins per say no external laser is able to deliver sufficient energy and that too uniformly circumferentially. This drawback was overcome with endo-luminal delivery of laser energy with an optical fiber. These are called as endovenous procedures and both radio frequency (RF) and lasers are being used as energy sources. The entire procedure is done under color doppler guidance. In RF a tiny catheter with five pronged tip is introduced in the vein. The four prongs are RF electrodes which open up like petals of a flower and come in contact with the vein wall. The central one is a thermocouple which continuously monitors the temperature and gives a feedback. The RF energy is directly delivered to the vein wall as the catheter is very slowly withdrawn. This causes vein wall damage and its immediate closure.

**Figure 16:**
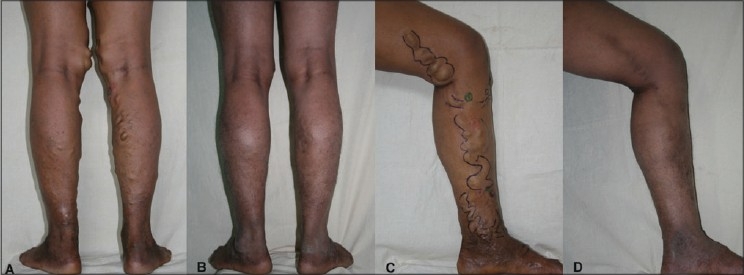
Varicose veins before and after EndoVenous Laser ablation with Diode 980 nm

In endovenous lasers initially Diode wavelengths like 808 nm, 810 nm and 840 nm were used. Now Diode 980 nm is being used and recently Nd: YAG 1320 nm is introduced. The Diode has absorption in blood while the latter is claimed to have more absorption in water contained in the cells of the vein wall causing it's closure. Both the RF and lasers have a definite learning curve with ease of it leaning towards lasers than the RF, because of its comparatively complicated gadgetry as compared to the simple 600 micron fiber of the lasers. In all endovenous procedures the GSV closure rate is around 90 to 95% [Figure 16].

### Side effects and complications

#### Therapeutic window

Usually every laser and light source has a therapeutic window, narrow or wide for a particular lesion or condition. As a rule of thumb, fluence in the therapeutic range is called as optimal fluence which gives a predictable clinical response and outcome. Sub optimal fluences are either non effective or at times have stimulating action while more than optimal fluences are clinically damaging.

Laser treated skin requires special care, regardless of the laser used or the lesion treated. As different lasers produce various skin responses, different post treatment regimens are required. Prior to treatment, sun exposure, tanning, retinoids and photo sensitizing drugs increase the chances of adverse effects and are contraindicated. After treatment the skin needs to be protected from heat, sun exposure, handling and infection. Laser treated skin generally tends to become drier and needs soothing and moisturizing lotions, creams or gels of aloe vera. It is a good policy to educate the clients on UV effects on skin and make them routinely use sun blocks with a minimum of 30 SPF (Sun Protecting Factor) till the last laser session. A habit cultivated will certainly help them in proper skin care.

#### Complications:

In laser treatment complications can be divided into immediate complications, late complications and sequelae. Immediate complications occur either immediately or within a few minutes or hours of laser treatment. These are usually related to excess fluence, occasionally related to improper technique and rarely related to an accident. Excess fluence leads to epidermal erythema, superficial burn or deep dermal burn with incident scarring depending on the extent of injury and pigmentary changes. It can be prevented or minimised by proper patient and lesion selection, proper parameter selection, test shots, stepping down on fluence during summer or when tanning is suspected, effective pre, intra and post laser cooling of the epidermis. However, in darker skin types this may not be evident immediately and therefore a test may be necessary.

#### Hair removal:

**Figure 17:**
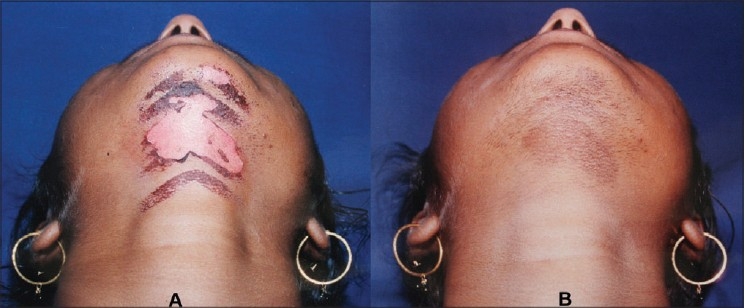
(A) Burns after 2nd HR treatment by IPL even with the longest 755 filter in a dark skin type. Photograph on 3rd day. This was a result of neglecting a warning in the form of a few very superficial brownish marks after 1st treatment with the same filter. In such a threshold situation, subsequent fluence should be decreased. Here it was rather increased by just 2 J/cm^2^ (B) Two months later hyper pigmentation has persisted. This acts as a hindrance to further treatments

In case of laser hair removal, particularly if plucking was practiced for a long time, folliculitis can occur. Other side effects are in the form of superficial burns either due to excess energy or energy intolerance. Erythema is most usual and transient but can remain for a few hours and occasionally for a day. Epidermal burns are more common over bony areas and are due to bouncing of the laser energy leading to coffee coloured crusting which usually clears within a day or two but is the cause of lot of anxiety to clients. Transient hyperpigmentation can ensue with more severe burns [Figure 17]. Usually it clears on its own but becomes a hindrance for the next laser session, therefore de-pigmenting creams are prescribed. Hypo-pigmentation is rare. Pigmentary changes are more seen in darker skin types. Paradoxical stimulation of more number of hair follicles in the area being treated or even in the adjacent areas which start growing longer and coarser hairs is a known entity. The possible explanations offered are, stimulating effect of the sub-optimal energy in the treatment area, scattering of the laser energy in adjacent dermis again acting by above mechanism and may be production and diffusion in the adjacent areas of some chemical mediators yet unknown to us. Whatever may be the cause, this complication apparently frightens the patient who needs assurance and confidence from the surgeon: “Its good that they are growing. More of them grow, more we will be able to destroy permanently in next sittings”.

#### Vascular:

**Figure 18:**
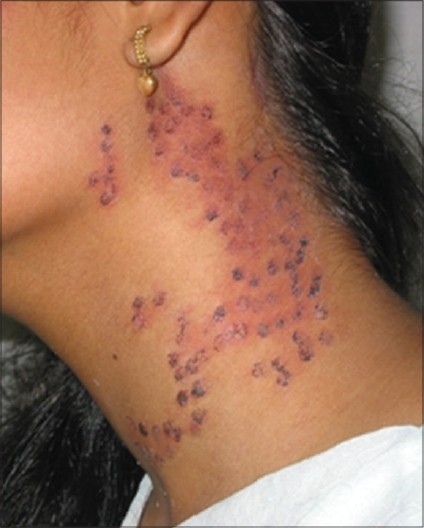
Purpuric spots after PDL 595 nm treatment of PWS. Usually they clear in a 5 to 7 days period

**Figure 19:**
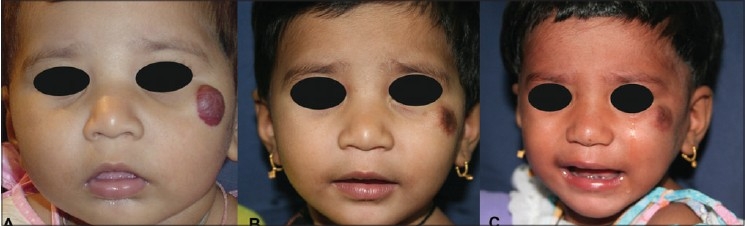
(A) Haemangioma over left cheek. (B) Hyper pigmentation following 5 Nd:YAG 1064 nm laser treatments. A single treatment with Q-Switched Nd:YAG overcame parental anxiety. (C) Pigment clearing after 2 months

Severe oedema and swelling, purpura, blisters, infection, crusting, hyper and hypo pigmentation and scarring can occur. In case of lesions like PWS, purpura and dry crusting are unavoidable [Figure 18]. Antiseptic creams should be used in the post laser period. Hyper pigmentation and hypo pigmentations can occur, both are temporary and do not need active treatment except in very anxious patients [Figure 19]. Very rarely it can be permanent and a mention of it should be made in the informed consent form.

#### Pigmeted areas:

**Figure 20:**
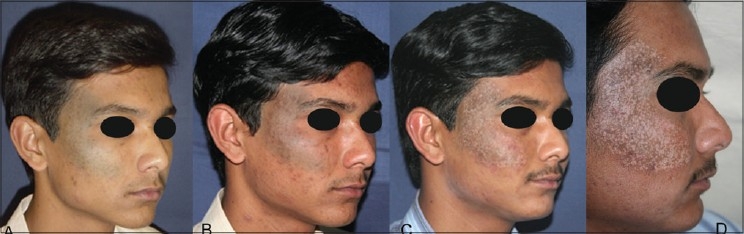
(A) Congenital Pigmented Nevus. Greenish hue indicates more deeper pigment (B) Clearing well with QS Nd:YAG 1064 nm laser (3 treatments). (C) Hypo-pigmentation appeared after 7^th^ treatment (D) Mottling increased with subsequent treatments and has persisted 3½ months after 12th treatment

As the therapeutic window narrows in pigmented lesions, chances of severe burns, hyper pigmentation and hypo pigmentation are more. Proper selection of the technology like Q-switching and strict adherence to the treatment and parameter protocols plays a very important role in their prevention. With the best of the above things, please remember that there is a vast difference between treating a pigmented lesion in lighter skin types and the similar lesion in darker skin type [Figure 20]. A safe policy is to start with the parameters at the bottom of the therapeutic window and make subtle increments only if there is no favourable response or the response is static. Protection from sun and depigmenting regimens acquire even more importance in these situations.

## EPILOGUE

Forty eight years are like a dot on the time scale of human evolution. Lasers are just that young. Looking at the different aspects of human life and the world as a community, the various positions and fields which lasers are occupying today are not less than miraculous. Medicine is no exception to this. Albeit slower but with the same miraculous results, laser technology has doubtlessly created a definite position of importance in therapeutic surgery and medicine.

Dr. Leon Goldman, MD, is aptly called the father of laser medicine. Immediately after invention of ruby laser in 1960 he used it for treating a variety of pigmented lesions and explored its effects on normal pigmented skin and in 1963 published first ever scientific article devoted to the subject of laser-skin interactions and the use of laser in medicine.[13] The ball was set rolling. Since then physicists and physicians all over the globe have worked hand in hand and have brought laser medicine and surgery to the present stage of glory. Unlike other non-medical fields here in medicine, lasers may still continue to act as an extension of the human senses. But who can predict about technology advancements? There may be a time in future, when this ‘human’ factor becomes meagre before the giant technology. And even if it happens, how would it matter? Ultimately, is it not the same human sense which has created the technology and will continue to refine it? Whether the technique or the technology, as long as it is used to alleviate human suffering, the joy in the mind using it should remain the same.
